# Supplementary material for: Both landscape heterogeneity and configuration determine Woodlarks (Lullula arborea) breeding territories
Source: PeerJ. 2021 Nov 19;9:e12476. doi: 10.7717/peerj.12476 (PMC8607927; doi:10.7717/peerj.12476)
Supplement: Supplemental Information 1 — Name, definition, and unit of measurement. [file peerj-09-12476-s001.docx]

| Variable | Definition | Calculated with/  Measured | Unit |
| --- | --- | --- | --- |
| Bare soil cover | Proportion of land covered by bare soil | FRAGSTATS | % |
| Grassland cover | Proportion of land covered by grassland | FRAGSTATS | % |
| Short cropland cover | Proportion of land covered by arable land with short vegetation (1-20 cm) | FRAGSTATS | % |
| Tall cropland cover | Proportion of land covered by arable land with tall vegetation (21-150 cm) | FRAGSTATS | % |
| Rough pasture cover | Proportion of land covered by rough pasture | FRAGSTATS | % |
| Forest cover | Proportion of land covered by forest | FRAGSTATS | % |
| Grove cover | Proportion of land covered by grove (cluster of trees) | FRAGSTATS | % |
| Residential area cover | Proportion of land covered by residential areas | FRAGSTATS | % |
| Dirt road cover | Proportion of land covered by dirt roads | FRAGSTATS | % |
| Asphalt road cover | Proportion of land covered by asphaltic streets | FRAGSTATS | % |
| Water cover | Proportion of land covered by other water bodies | FRAGSTATS | % |
| Mean height grassland | Mean height of grassland vegetation, measured at several points according to the size of the polygon | field | cm |
| Mean height short cropland | Mean height of arable land with short vegetation (1-20 cm), measured at several points according to the size of the polygon | field | cm |
| Mean height tall cropland | Mean height of arable land with tall vegetation (21-150 cm), measured at several points according to the size of the polygon | field | cm |
| Mean height rough pasture | Mean height of rough pasture vegetation, measured at several points according to the size of the polygon | field | cm |
| Mean height vegetation track lanes dirt road | Mean height of vegetation track lanes dirt road, measured at several points according to the size of the polygon | field | cm |
| Mean height forest | Estimated mean height of forest, measured at several points according to the size of the polygon | field | m |
| Mean height grove | Estimated mean height of grove (cluster of trees), measured at several points according to the size of the polygon | field | m |
| DBH forest | Mean diameter at breast height of forest, measured at several points according to the size of the polygon | field | m |
| DBH grove | Mean diameter at breast height of grove (cluster of trees), measured at several points according to the size of the polygon | field | m |
| Length dirt road | Length of dirt roads within the plot | ArcGIS | m |
| Length asphalt road | Length of asphaltic roads within the plot | ArcGIS | m |
| Length electricity line | Length of electricity lines within the plot | ArcGIS | m |
| Length hedgerow | Length of hedgerows within the plot | ArcGIS | m |
| Distance to dirt road | Distance from the centre of the plot to the closest dirt road | ArcGIS / field | m |
| Distance to electricity line | Distance from the centre of the plot to the closest electricity line | ArcGIS / field | m |
| Distance to hedgerow | Distance from the centre of the plot to the closest hedgerow | ArcGIS / field | m |
| Distance to forest | Distance from the centre of the plot to the closest forest | ArcGIS / field | m |
| Distance to groves | Distance from the centre of the plot to the closest grove (cluster of trees) | ArcGIS / field | m |
| Landscape level |  |  |  |
| Patch density (PD) | Overall patch density. Computed as the number of all patches in the landscape divided by total area of the plot | FRAGSTATS | #/100 ha |
| Landscape shape index (LSI) | Measures the overall geometric complexity and gives information about dispersion of patches in the plot | FRAGSTATS | unitless |
| Proximity index (PROX_MN) | Calculates the degree of patch isolation by considering both the size and the proximity to all patches in the plot | FRAGSTATS | m |
| Contagion index (CONTAG) | Measures both patch type interspersion (i.e., the intermixing of different patch types) and patch dispersion (i.e., the spatial distribution of a patch type) in the plot | FRAGSTATS | unitless |
| Simpson diversity index (SIDI) | Calculates the landscape diversity within the plot | FRAGSTATS | unitless |
| Class level |  |  |  |
| PD bare soil | Patch density of bare soil patches in the plot | FRAGSTATS | #/100 ha |
| PD grassland | Patch density of grassland patches in the plot | FRAGSTATS | #/100 ha |
| PD short cropland | Patch density of arable land with short vegetation (1-20 cm) patches in the plot | FRAGSTATS | #/100 ha |
| PD tall cropland | Patch density of arable land with tall vegetation (21-150 cm) patches in the plot | FRAGSTATS | #/100 ha |
| PD rough pasture | Patch density of rough pasture patches in the plot | FRAGSTATS | #/100 ha |
| PD forest | Patch density of forest patches in the plot | FRAGSTATS | #/100 ha |
| PD grove | Patch density of grove (cluster of trees) patches in the plot | FRAGSTATS | #/100 ha |
| PD residential area | Patch density of residential area patches in the plot | FRAGSTATS | #/100 ha |
| PD dirt road | Patch density of dirt road patches in the plot | FRAGSTATS | #/100 ha |
| PD asphalt road | Patch density of asphaltic street patches in the plot | FRAGSTATS | #/100 ha |
| PD water | Patch density of water bodies in the plot | FRAGSTATS | #/100 ha |
| LSI bare soil | Measures the disaggregation or aggregation of arable land without vegetation patches in the plot | FRAGSTATS | unitless |
| LSI grassland | Measures the disaggregation or aggregation of grassland patches in the plot | FRAGSTATS | unitless |
| LSI short cropland | Measures the disaggregation or aggregation of arable land with short vegetation (1-20 cm) patches in the plot | FRAGSTATS | unitless |
| LSI tall cropland | Measures the disaggregation or aggregation of arable land with tall vegetation (21-150 cm) patches in the plot | FRAGSTATS | unitless |
| LSI rough pasture | Measures the disaggregation or aggregation of rough pasture patches in the plot | FRAGSTATS | unitless |
| LSI forest | Measures the disaggregation or aggregation of forest patches in the plot | FRAGSTATS | unitless |
| LSI grove | Measures the disaggregation or aggregation of grove (cluster of trees) patches in the plot | FRAGSTATS | unitless |
| LSI residential area | Measures the disaggregation or aggregation of residential area patches in the plot | FRAGSTATS | unitless |
| LSI dirt road | Measures the disaggregation or aggregation of dirt road patches in the plot | FRAGSTATS | unitless |
| LSI asphalt road | Measures the disaggregation or aggregation of asphaltic street patches in the plot | FRAGSTATS | unitless |
| LSI water | Measures the disaggregation or aggregation of other used patches in the plot | FRAGSTATS | unitless |
